# Supplementary material for: Content-rich biological network constructed by mining PubMed abstracts
Source: BMC Bioinformatics. 2004 Oct 8;5:147. doi: 10.1186/1471-2105-5-147 (PMC528731; doi:10.1186/1471-2105-5-147)
Supplement: Additional File 2 — The original results of the above study (non-essential files are deleted to keep the file size under the limit set by BMC bioinformatics). [file 1471-2105-5-147-S2.bz2 › chilibotAdditionalFile2/dip10/90ID7962001E327/html/RECO_SSB.html]

 


 **RECO** and **SSB** 
  
Found 13 abstracts in PubMed, retrieved 10.  
 

 What does Google say? 
 PDF only 
| .edu only 

---

**Interactive relationship** (e.g. stimulation, inhibition, etc)

**Inhibitory relationship**- Interactions involving  **RecO**  and RecR overcome the inhibition of RecA by single stranded DNA binding protein  [ **SSB** ] .  Ref: 7962001 J Biol Chem, 1994
- RecR protein, which is known to stimulate  **RecO**  protein to facilitate  **SSB**  protein displacement by RecA protein, inhibits annealing by  **RecO**  protein, suggesting that RecR protein may regulate the choice between the DNA strand invasion versus annealing pathways.  Ref: 12438681 Proc Natl Acad Sci U S A, 2002
- However,  **RecO**  and RecR can act together as accessory factors for RecA to overcome this inhibition by  **SSB**  Umezu, K.  Ref: 7962001 J Biol Chem, 1994
**Neutral relationship**- The interaction between  **RecO**  and  **SSB**  is stronger than the  **RecO**  RecR interaction.  Ref: 7962001 J Biol Chem, 1994
- i  **RecO**  physically interacts with both RecR and  **SSB** .  Ref: 7962001 J Biol Chem, 1994
- These data suggest that one role of RecF is to stabilize the interaction of RecR with  **RecO**  in the presence of  **Ssb**  protein.  Ref: 8962075 Proc Natl Acad Sci U S A, 1996
- First,  **RecO**  and RecR bind to  **SSB** .ssDNA complexes and modify their structure to allow RecA to nucleate on them efficiently.  Ref: 7962001 J Biol Chem, 1994
- These data suggested that  **RecO**  mediates the interactions of RecF protein with RecR and with  **Ssb**  proteins.  Ref: 8962075 Proc Natl Acad Sci U S A, 1996
- Optimal activity for  **RecO**  protein occurs after formation of a 1 1 complex with  **SSB**  protein.  Ref: 12438681 Proc Natl Acad Sci U S A, 2002
- Incubation of RecF,  **RecO** , RecR, and  **Ssb**  proteins resulted in the formation of RecF  **RecO**   **Ssb**  complexes ; i.e., RecR was excluded.  Ref: 8962075 Proc Natl Acad Sci U S A, 1996
- The resulting  **RecO** .RecR. **SSB** .ssDNA complexes were more active in RecA mediated joint molecule formation than were  **SSB** .ssDNA complexes.  Ref: 7962001 J Biol Chem, 1994
- Preincubation of RecF,  **RecO** , and RecR proteins prior to addition of  **Ssb**  protein resulted in the formation of complexes consisting of RecF,  **RecO** , RecR, and  **Ssb**  proteins.  Ref: 8962075 Proc Natl Acad Sci U S A, 1996
- The products of the recF,  **recO** , and recR genes are thought to interact and assist RecA in the utilization of single stranded DNA precomplexed with single stranded DNA binding protein  [ **SSB** ]   **Ssb**  during synapsis.  Ref: 8962075 Proc Natl Acad Sci U S A, 1996

**Non-interactive relationship** (e.g. studied together, co-existance, homology, etc.)

- Using immunoprecipitation, size exclusion chromatography, and  **Ssb**  protein affinity chromatography in the absence of any nucleotide cofactors, we have obtained the following results i RecF interacts with  **RecO** , ii RecF interacts with RecR in the presence of  **RecO**  to form a complex consisting of RecF,  **RecO** , and RecR RecF  **RecO**  RecR.  Ref: 8962075 Proc Natl Acad Sci U S A, 1996
- iv When RecA presynaptic filaments were formed in the presence of  **SSB** ,  **RecO** , and RecR, the protein DNA complexes obtained contained 70% of the amount of RecA required to saturate ssDNA.  Ref: 7962001 J Biol Chem, 1994
- ii  **RecO**  and RecR do not remove  **SSB**  from  **SSB** .ssDNA complexes, but instead bind to these complexes.  Ref: 7962001 J Biol Chem, 1994
- Finally, examples are shown for in vitro reactions involving DNA, RecA,  **RecO** , RecF, RecR, and  **SSB**  that lead to a further understanding of recombinational repair.  Ref: 11098470 Mol Biotechnol, 2000
- We discuss these results to explain how the RecF  **RecO**  RecR complex functions as an anti  **Ssb**  factor.  Ref: 8962075 Proc Natl Acad Sci U S A, 1996
- To elucidate the mechanism that underlies this process, we examined protein protein interactions between RecA, RecF,  **RecO** , RecR, and  **SSB** , and characterized the structure and activity of the ssDNA complexes formed with different combinations of these proteins.  Ref: 7962001 J Biol Chem, 1994
- iii RecA can nucleate on the  **RecO** .RecR. **SSB** .ssDNA complexes more efficiently than on  **SSB** .ssDNA complexes.  Ref: 7962001 J Biol Chem, 1994
- iii RecF interacts with  **Ssb**  protein in the presence of  **RecO** .  Ref: 8962075 Proc Natl Acad Sci U S A, 1996
- An increase of intracellular  **RecO** , which may be due to the increased  **recO**  gene copies or to the induction of  **RecO**  synthesis, increased the UV resistance of recA, recF, and  **ssb**  cells, but did not increase the UV resistance of uvrB, uvrB recF, uvrB recA and uvrB  **ssb**  cells.  Ref: 8538645 Mutat Res, 1996
